# Editorial: Methods and protocols in obstetric and pediatric pharmacology: 2022

**DOI:** 10.3389/fphar.2023.1205963

**Published:** 2023-05-11

**Authors:** Kathleen M. Job, Jessica K. Roberts, Catherine M. Sherwin

**Affiliations:** ^1^ Division of Clinical Pharmacology, Department of Pediatrics, School of Medicine, The University of Utah, Salt Lake City, UT, United States; ^2^ Simulations Plus, Inc., Cognigen Division, Lancaster, CA, United States; ^3^ Department of Pediatrics, Boonshoft School of Medicine, Wright State University, Dayton, OH, United States

**Keywords:** pregnancy pharmacology, maternal-fetal pharmacology, pediatric pharmacology, experimental methods, experimental approach

## Background

Generally, the techniques and procedures employed in obstetric and pediatric pharmacology are created to ensure that medicines are administered to these vulnerable groups safely and efficiently. Working closely with medical professionals who are competent in this field is vital (Zimmerman et al., 2015; Obstetric and Pediatric Pharmacology and Therapeutics Branch, 2022). Obstetric and pediatric pharmacology procedures and methods must be appropriate for pregnant women, new mothers, and children to utilize drugs safely and effectively (Dathe and Schaefer, 2019).

Most obstetric pharmacology research considers pregnant women’s and developing fetuses’ safety. As a result, medications that are safe for use in adults may not be safe during pregnancy because they could cross the placenta and harm the fetus (Haas et al., 2009). Hence, thoroughly weighing the advantages and disadvantages of any drug before administering it to a pregnant woman is one of the crucial techniques employed in obstetric pharmacology. This involves considering the pregnancy stage, the medication dosage, and possible side effects to both the mother and the fetus (Sachdeva et al., 2009).

The safety and effectiveness of medications in children, who may have different physiological and metabolic features than adults, are the main concerns in pediatric pharmacology (Batchelor and Marriott, 2015). The two areas of drug development where these considerations are evaluated include pharmacokinetic studies, and efficacy and safety studies (Phase 2 clinical trials). Pharmacokinetic studies provide information on the absorption, distribution, metabolism, and excretion of medications in pregnant women, children, and young children. The appropriate dosage of a drug for each population must be determined using this information while accounting for age and weight, among other factors (Field and Behrman, 2004). Furthermore, formulation requires special consideration in pediatric patients. Because some medications are not yet accessible in pediatric-specific formulations, pediatric pharmacologists may need to collaborate with manufacturers to create novel formulations (Juarez-Hernandez and Carleton, 2022). The safety and effectiveness of drugs are examined concerning pregnant mothers, fetuses, newborns, and children in Phase 2 clinical trials. These studies are planned to reduce dangers to research participants and guarantee reliable and accurate results (Batchelor and Marriott, 2015). Monitoring adverse events is essential for spotting potential safety issues with drugs used in children, infants, and pregnant women (Shakhnovich et al., 2019). These studies, taken together with the pharmacokinetic data, aid in final dose selection for each individual population.

## Objective

The goal of this editorial is to present the most up-to-date experimental approaches and methods for basic pharmacology research in the specialty section of Obstetrics and Pediatrics of Frontiers.

1. Experimental and clinical progress of in-utero hematopoietic cell transplantation therapy for congenital disorders (Shi et al.).

Fetal interventions for congenital diseases have significantly advanced. While historically, myeloablation has been used to treat these diseases postnatally, an alternative to postnatal treatment is *in utero* hematopoietic cell transplantation. *In utero* hematopoietic cell transplantation (IUHCT) enables doctors to treat disorders before birth instead of postnatal therapy. Shi et al. review the obstacles to engraftment and give possible solutions.

Attempts have been made over the past few decades to remove obstacles that stand in the way of effective IUHCT in healthy recipients and to enhance the engraftment of donor cells without graft-versus-host disease (GVHD). These tactics can be divided into three groups: Giving donor cells a competitive edge, expanding donor-friendly habitats, and getting beyond immunological barriers are the three objectives. For example, hematopoietic stem cells can be extracted from adult bone marrow, fetal liver, and umbilical cord blood, but more research is required to determine whether or not amniotic fluid stem cells can engraft in allogeneic or xenogeneic animal models.

Some promising techniques to enhance the engraftment of donor cells may be implemented with a better understanding of the maternal and fetal immunological barriers. Engraftments may be enhanced by aiming for an initial chimerism threshold of >1.8% with host natural killer cell tolerance. For instance, enhancing donor cell chimerism following IUHCT might be possible by decreasing host hematopoietic stem cells (HSC) niches with a silver bullet. Congenital hematological, genetic, and immunological problems are all treatable using IUHCT. The only pregnancies this therapy has been effective so far are those with severe combined immunodeficiency. Before IUHCT may be utilized as a therapeutic substitute for a particular ailment, several obstacles must be cleared. However, it appears unlikely to achieve a therapeutic degree of engraftment in the human fetus without developing certain myeloablative medications or alternatives.

2. Using intranasal dexmedetomidine with buccal midazolam for magnetic resonance imaging sedation in children: A single-arm prospective interventional study (Li et al.)

Magnetic resonance imaging (MRI) in recalcitrant children requires an acceptable level of sedation. Due to its rapid onset and appropriate drowsiness, propofol has historically been the primary sedative for pediatric MRIs, but issues with dose-dependent respiratory depression and hemodynamic instability have been brought up.

There is no established best practice for non-parenteral sedation during MRI. This prospective, interventional trial determined whether giving children receiving an MRI the combination of buccal midazolam and intranasal dexmedetomidine was safe and effective. With the earliest sedative regimens, the success rate for sedation was 95.3%. The only other risk factor deemed statistically significant was a history of unsuccessful sedation. In 35 (6.6%) and 12 (2.3%) individuals, hypotension and bradycardia occurred. A brief drop in blood pressure below 30% of the age group’s normal range occurred in four patients (0.75%). No children needed oxygen support or medicine administration.

Combining low-dose buccal midazolam with intranasal dexmedetomidine administration was linked with a high success rate in short duration MRI exams. Specifically, this non parenteral sedation regimen demonstrated smooth hemodynamic condition, no significant adverse events, and appropriate sedation in 97.7% of children between 1 month and 10 years. The benefits of this non-parenteral regimen over intravenous access include simple operation and a strong safety record.

3. Potential serum biomarkers associated with premature rupture of fetal membranes in the first trimester (An et al.)

A common and severe obstetric complication known as premature rupture of the fetal membranes (PROM) has a higher risk of adverse outcomes for mothers and fetuses. An accurate and fast approach to PROM prediction is required to protect the mother and fetus’s safety. The study published by An et al. provides insight into early pregnancy PROM prediction.

The causes of PROM are multifactorial and include membrane structure, inflammation, apoptosis, oxidative stress, and other elements. Glycerophospholipids are essential to cell membrane structure and play a key role in numerous biological processes. Correlation analysis was used to identify the temporal intensity profiles of the 13 PROM-related lipid metabolites altered during the first trimester. Significant increases or decreases in these metabolites occurred and tended to group. Three upregulated metabolites linked with the glycerophosphocholines (PC) O-40:6 and five of the six downregulated glycerophospholipid metabolites showed positive intra-correlation and were significantly different between PROM and control cohorts. According to this study, correlations between putative biomarkers, PCs, and glycerophosphoethanolamines (PEs) comprised the most significant cluster. This raises the notion that glycerophospholipids may be closely related to the altered metabolome dynamics seen during early pregnancy associated with PROM, although correlation does not imply causality. Additionally, WISH cell viability was inhibited by a ceramide, Cer 40:0; O2, suggesting that cell survival or proliferation may be a potential cause. Furthermore, this study clarified how to anticipate PROM in the first trimester of pregnancy and how to comprehend its underlying mechanism. As a result of this analysis, a composite model was created using four metabolites to forecast the possibility of PROM in the early stages of pregnancy.

4. The effect of dietary fiber supplement on prevention of gestational diabetes mellitus in women with pre-pregnancy overweight/obesity: A randomized controlled trial (Zhang et al.)

The study’s rationale was that increasing dietary fiber consumption during pregnancy is crucial for lowering the risk of metabolic problems. In order to determine whether intervention with a dietary fiber supplement, as opposed to standard prenatal care during pregnancy, would reduce the risk of gestational diabetes mellitus (GDM) and improve maternal pregnancy outcomes, the authors planned and carried out a randomized controlled trial in pre-pregnancy overweight/obese women. The study was conducted in China, where pregnant Chinese women consume 14.9 g of fiber on average daily, far less than the 25 g recommended daily consumption.

When compared to the control group, the fiber supplement group had a considerably lower incidence of GDM. However, 46.7% of the women in the fiber supplement group experienced excessive weight increase (total weight gain >11.5 kg for overweight and >9.0 kg for obesity). None of the women randomly assigned to the fiber supplement group delivered prematurely. According to the authors, the triglyceride (TG) and triglyceride to high density lipid-cholesterol (TG/HDL-C) ratio levels in the intervention group were considerably higher than those in the control group.

Women in the intervention group gave birth to babies significantly younger than those in the control group. Due to the unicentric nature of the study and the tiny sample sizes in the two groups, it may be difficult to determine accuracy. The incidence of pregnancy-induced hypertension, preeclampsia, polyhydramnios, cesarean section, macrosomia, and neonatal distress respiratory syndrome was not shown to be decreased by dietary fiber in the study. Also, consuming more dietary fiber when pregnant may lower the risk of GDM, excessive weight gain, and preterm birth, but it does not improve blood lipids. This is true even for pregnant women who were overweight or obese before becoming pregnant.

The papers mentioned above highlight cutting-edge experimental approaches to examining fundamental issues in obstestric and pediatric pharmacology. Although the usual techniques employed in these sectors are comparable to those employed in other areas of pharmacology, there are some notable variations (Sachdeva et al., 2009).

The study of medications administered to pregnant women is one of the most understudied areas in clinical pharmacology and drug development. Furthermore, pediatric studies on disease-specific treatments are lacking and, many times, new clinical candiates are killed during adult clinical trials without ever evaluating possible effectiveness in children. While the complexity of maternal and pediatric methods and therapeutic investigation protocols must cover everything from the treatment and drug development of obstetric and breastfeeding conditions to the treatment of premature neonates, we submit it is unethical to ignore these vulnerable populations because of these complexities.
